# Wedged gel pad for bowel manipulation during MR-guided high-intensity focused ultrasound therapy to treat uterine fibroids: a case report

**DOI:** 10.1186/s40349-018-0116-4

**Published:** 2018-11-28

**Authors:** Teija Sainio, Gaber Komar, Jani Saunavaara, Visa Suomi, Kirsi Joronen, Antti Perheentupa, Antti Viitala, Roberto Blanco Sequeiros

**Affiliations:** 1 Medical Imaging Centre of Southwest Finland, University of Turku and Turku University Hospital, Turku, Finland; 2 Department of Medical Physics, Division of Medical Imaging, Turku University Hospital, Turku, Finland; 3 Department of Obstetrics and Gynecology, Turku University Hospital, Turku, Finland

**Keywords:** Gel pad, Uterine fibroid, Bowel repositioning, MR-guided high-intensity focused ultrasound

## Abstract

**Background:**

Magnetic resonance guided high-intensity focused ultrasound (MR-HIFU) therapy is not feasible in all patients with uterine fibroids because of limiting anatomical factors such as scar tissue, bowel loops or other obstacles in the sonication path. These may prevent the treatment or limit the treatment window, and therefore, also the volume where HIFU therapy can be delivered. Bowel loops present a particular problem because of bowel gas bubbles and hard particles which may cause reflection or absorption of ultrasound energy, potentially leading to thermal damage and even bowel perforation. Most commonly used techniques for bowel repositioning are bladder and/or rectum filling but these are not always sufficient to reposition the bowel loops. With more efficient bowel repositioning technique, the number of eligible patients for MR-HIFU treatment could be increased, and therapy efficacy be improved in cases where bowel loops limit the treatment window.

**Case presentation:**

A wedged exterior gel pad was used in two patients presented with in total of four symptomatic fibroids undergoing MR-HIFU treatment when bladder and/or rectum filling was not sufficient to reposition the bowel loops. No severe adverse effects were observed in these cases. The non-perfused volume ratios (NPVs) immediately after treatment were 86% and 39% for the first patient, and 3% for the second patient.

**Conclusions:**

Our preliminary experience suggests that the use of a wedged gel pad during MR-HIFU treatment could be an effective tool to manipulate the bowels in cases where the bladder and/or rectum filling is not sufficient to reposition the bowel loops. A wedged gel pad could also be used in other situations to achieve better treatment coverage to the uterine fibroid.

## Background

Uterine fibroids, also known as leiomyomas or myomas, are the most frequent benign tumors affecting women in premenopausal age [1, 2]. Uterine fibroids can cause symptoms including severe menorrhea, urinary incontinence, dysmenorrhea, and infertility which can have a huge impact to women’s quality of life [3–6]. Current, conventional treatment options for symptomatic uterine fibroids are medical, surgical, and uterine arterial embolization (UAE) which can have major side effects or are invasive [6].

Magnetic resonance-guided high-intensity focused ultrasound (MR-HIFU) therapy is a promising noninvasive treatment method for uterine fibroids with good adverse event profile [7]. In MR-HIFU uterine fibroid therapy the ultrasound waves are focused into the body targeting fibroid tissue, to form a localized area of high-intensity ultrasound (HIU), inducing rapid temperature elevation. Heat generation is due to absorption of the ultrasound waves in tissue, therefore acoustic energy is transformed to heat. Heating the tissue above 57^∘^C induces, e.g., coagulative necrosis and irreversible tissue damage in seconds, while surrounding tissue remains viable [8, 9]. At the moment, uterine fibroids are one of the most commonly MR-HIFU -treated tumors. In addition, HIFU’s clinical efficacy in the treatment of uterine fibroids has been widely shown [10–15].

However, not all patients are suitable for MR-HIFU therapy. For reasons such as excessively thick subcutaneous fat layer (> 4 cm), large fibroid (> 10 cm), location of fibroid (fibroid is too deep, > 10 cm from the skin) or fibroid tissue type is not favorable (Funaki classification). Funaki et al. [16] showed that the efficacy of MR-HIFU treatment correlates with the signal intensity of T2-weighted magnetic resonance images and classified fibroids into 3 types based on the signal intensity of T2-weighted magnetic resonance images as follows: type 1, low intensity; type 2, intermediate intensity; type 3, high intensity. Funaki type 1 and type 2 fibroids are suitable candidates for MR-HIFU treatment, whereas type 3 fibroids are not.

One major factor negatively affecting HIFU treatment feasibility is bowel loops between the abdominal wall and the uterine fibroid. Since, the ultrasound beam focusing is implemented by a concave ultrasound transducer that consists of multiple transducer elements. The ultrasound beam shape is a conical being wider at the base. This conical shape of the beam may present a limitation in the uterine fibroid treatment delivery due to overlying bowel gas bubbles and hard particles within the lumen which may reflect or absorb the ultrasound energy in unpredictable ways. This may potentially lead to unintended thermal damage, and at worst, bowel perforation that requires surgical intervention [17–21]. A safe acoustic window can be established in some cases with beam shaping and/or tilting, or by manipulating bowel loops with bladder and/or rectum filling [22].

Previous studies [17, 18, 23] have reported that 14-74% of patients requesting MR-HIFU are good candidates for the treatment, and in 7-13% of cases bowels were partially or completely obstructing the acoustic window. Usually, the bowel loops can be repositioned with bladder and/or rectum filling but these are not always sufficient to reposition the bowel loops [22, 24]. Kim et al. [24] reported that bladder and/or rectum filling technique is not effective in repositioning bowels in 5% of bowel obstruction cases. With an alternative or adjuvant bowel repositioning technique, the number of patients suitable for MR-HIFU treatment could be increased, and better treatment results could be achieved in cases where the bowel loops limit the treatment window.

Hesley et al. [25] reported that in some cases, a convex gel pad was helpful in displacing the bowel loops away from the treatment window during HIFU therapy. However, a disadvantage of the method was that the use of a convex gel pad resulted in a considerably longer distance between the transducer and targeted tissue, limiting the reach of HIFU. The study did not assess the safety and feasibility of convex gel pad for bowel displacement during HIFU therapy.

The purpose of this case report is 1) to introduce a novel way of repositioning the bowel loops positioned between the abdominal wall and the uterine fibroid by using a wedge shaped gel pad, and 2) to discuss the potential benefits and drawbacks of this technique from the aspect of safety and feasibility.

## Case presentation

### Materials and methods

Patients were recruited to our clinical trial (clinicaltrials.gov identifier NCT02914704) based on their wish not to undergo surgical treatment among other criteria. Patients underwent MR imaging protocols for screening, HIFU therapy, and 3-month follow-up as shown in Table 1.

**Table 1:** MRI imaging protocols for screening, HIFU therapy and 3-month follow-up

Examination	Sequence type	Purpose	Imaging plane	TR (ms)	TE (ms)	FA (^∘^)	Section thickness (mm)	FOV (mm)
Screening	T2W TSE	Uterine fibroid	Sagittal	4844	95	90	3	240x240
	T2W TSE	Uterine fibroid	Axial	3845	80	90	4	300x300
	T1W	Uterine fibroid	Sagittal	5.2	2.6	7	3	345x250
	DWI	Uterine fibroid	Axial	3733	83	90	5	375x290
	CE-T1W	Uterine fibroid	Sagittal	5.2	2.6	7	3	345x250
Treatment	FFE	Air bubble assessment	Coronal	15	12	10	2	280x280
	T2W TSE	Treatment planning	Sagittal	1550	150	90	1.6	250x250
	FFE-EPI	Temperature monitoring	Coronal/Sagittal	37	19.5	19	7	400x400
	CE-T1W	Non-perfused volume	Coronal	5.2	2.6	7	3	345x250
3-month follow-up	T2W TSE	Uterine fibroid volume	Sagittal	4844	95	90	3	240x240
	T2W TSE	Uterine fibroid volume	Axial	3845	80	90	4	300x300
	CE-T1W	Non-perfused volume	Sagittal	5.2	2.6	7	3	345x250

HIFU therapy was performed using an extracorporeal, clinical tabletop MR-HIFU system (Sonalleve V2 MR-HIFU system, Profound Medical Inc., Mississauga, Canada) in combination with a 3.0 T clinical MR scanner (Ingenia, Philips Healthcare, Best, The Netherlands). Immediately prior to the therapy, lower abdomen was depilated and a Foley catheter was inserted. Premedication (1 g paracetamol, 800 mg ibuprofen and 5 mg diazepam) was administered to reduce possible pain and discomfort during the treatment. The patient was positioned on the Sonalleve table in prone position and acoustic coupling between skin and the device was achieved with degassed and deionized water. The Sonalleve table was then advanced into the magnet bore and 3D T2-weighted TSE images were acquired and transferred to therapy delivery console for treatment planning. Based on these images, a safe sonication path was ensured by excluding any interleaving bowel loops or, if necessary, by repositioning them.

In case of bowel obstruction our protocol is 1) bladder filling (till discomfort) 2) rectum filling if necessary 3) bladder emptying. At each point we try to establish sufficient coverage (> 50% of the fibroid) to the fibroid with beam shaping or tilting. Due to natural limitations of these techniques, we are sometimes forced to apply the herein described wedged gel pad and in some cases also vaginal pessary in order to improve the position of the uterine cervix. We have used wedged gel pad in total of three cases and have achieved a satisfactory reposition of the bowel loops in all three cases.

**Fig. 1:**
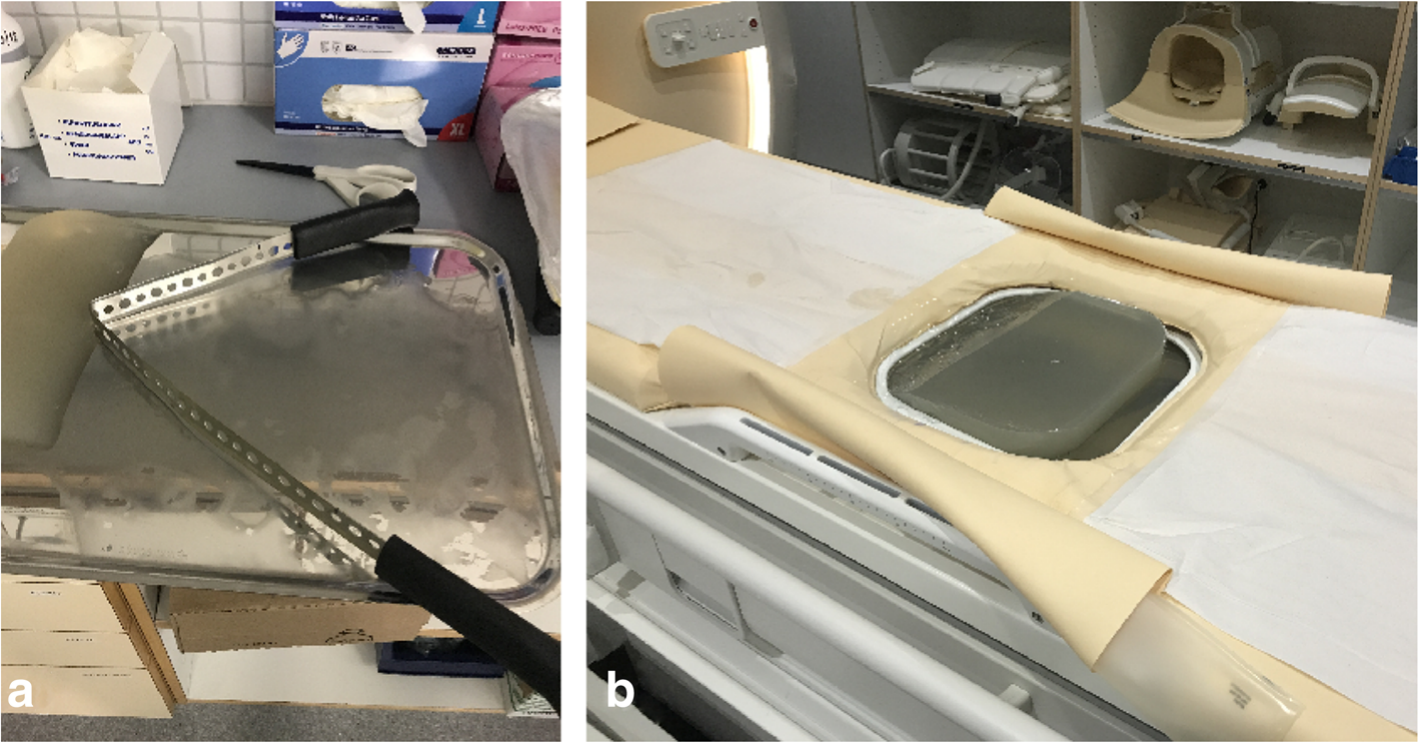
**a**) The blade that can be used to modify gel pad and **b**) produced wedged gel pad on the treatment window

A gel pad (27.5 × 27.5 × 4.0 cm^3^, Aquaflex, Parker Laboratories Inc., Germany) was sculpted into wedged shape using a sharp, shapeable blade manufactured by our technical division (Fig. 1). With the use of a sharp blade the cutting surface was smooth preventing air bubbles getting trapped between the skin and the gel pad. Shaping of the gel pad can also be performed with other type of cutting device that results in a smooth cutting surface in order to prevent air pockets created by surface irregularities. The sloping edge was placed caudal to the bowels and reached from side to side. The cutting was done approximately in 45 degrees angle. Degassed water was added between the gel pad and acoustic window, and ultrasound gel-water (1:1) solution was added between the gel pad and abdominal skin to ensure good acoustic coupling.

**Fig. 2:**
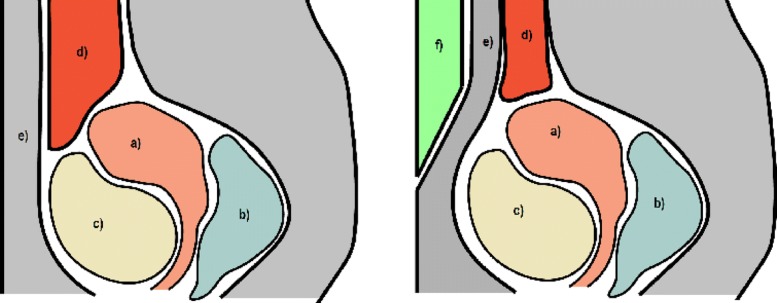
A diagrammatic presentation of the anatomy before and after wedged gel pad placement, where a) uterus with myoma, b) US-gel filled rectum, c) saline filled bladder, d) intestines moving within the abdominal cavity, e) abdominal wall and f) gel pad compressing the abdominal cavity, restricting the anterior-posterior space where intestines may move

The wedged gel pad, due to its shape, creates a force in the anteroposterior direction as well as in caudocranial direction. This increases the pressure in the cranial part of the lower abdomen (while not affecting the location of pelvic structures) whereby the bowel loops located between the uterus and the abdominal wall are displaced in the cranial direction (Fig. 2). The wedged shape not only facilitates this dislocation but also allows for a better seal between the gel pad and the skin on the other hand and smoother transition between the gel pad and the transducer surface eliminating the problem of air bubbles and skin fold.

#### Case 1

**Fig. 3:**
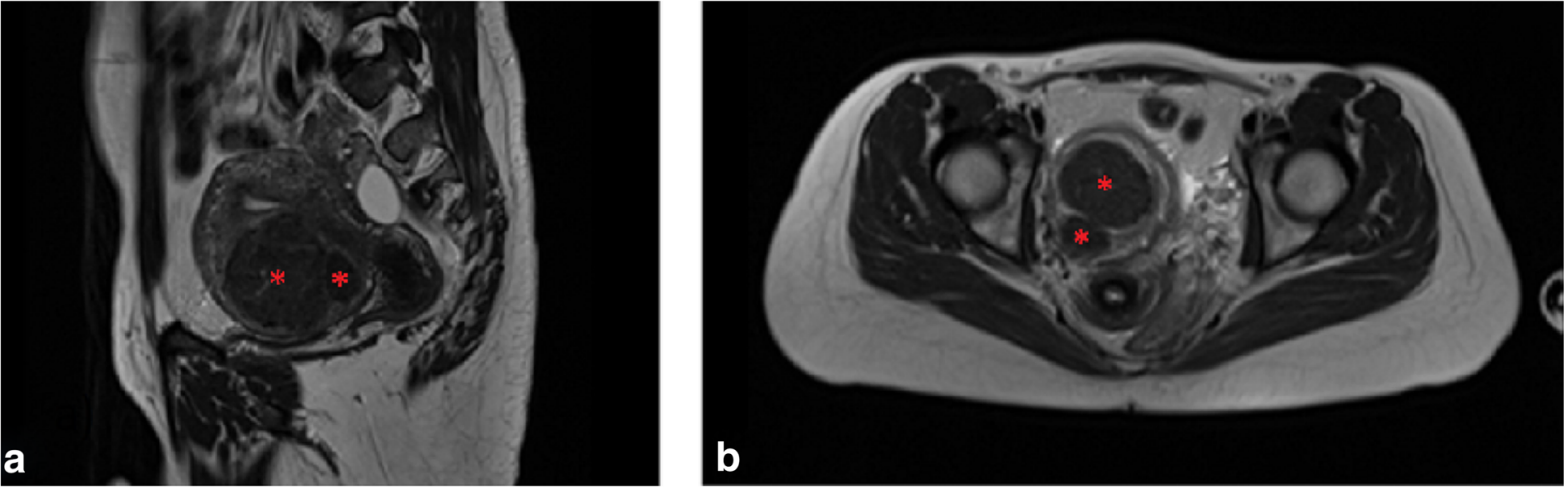
T2W screening images of uterine fibroids (red asterisks) in **a**) the sagittal and **b**) axial plane

A 33-year-old woman of African background with three symptomatic intramural uterine fibroids in the anterior wall which caused deformation of the uterine cavity with two years of unsuccessful pregnancy attempts. Patient had previously underwent a laparoscopic enucleation of uterine fibroid in the posterior wall. The fibroids were classified as Funaki type I based on T2W images, and contrast-enhanced T1W images were assessed. Fibroid sizes were 5.0 × 4.6 × 4.8 cm^3^, 2.5 × 1.3 × 2.0 cm^3^, and 2.8 × 1.9 × 2.4 cm^3^ (Fig. 3).

**Fig. 4:**
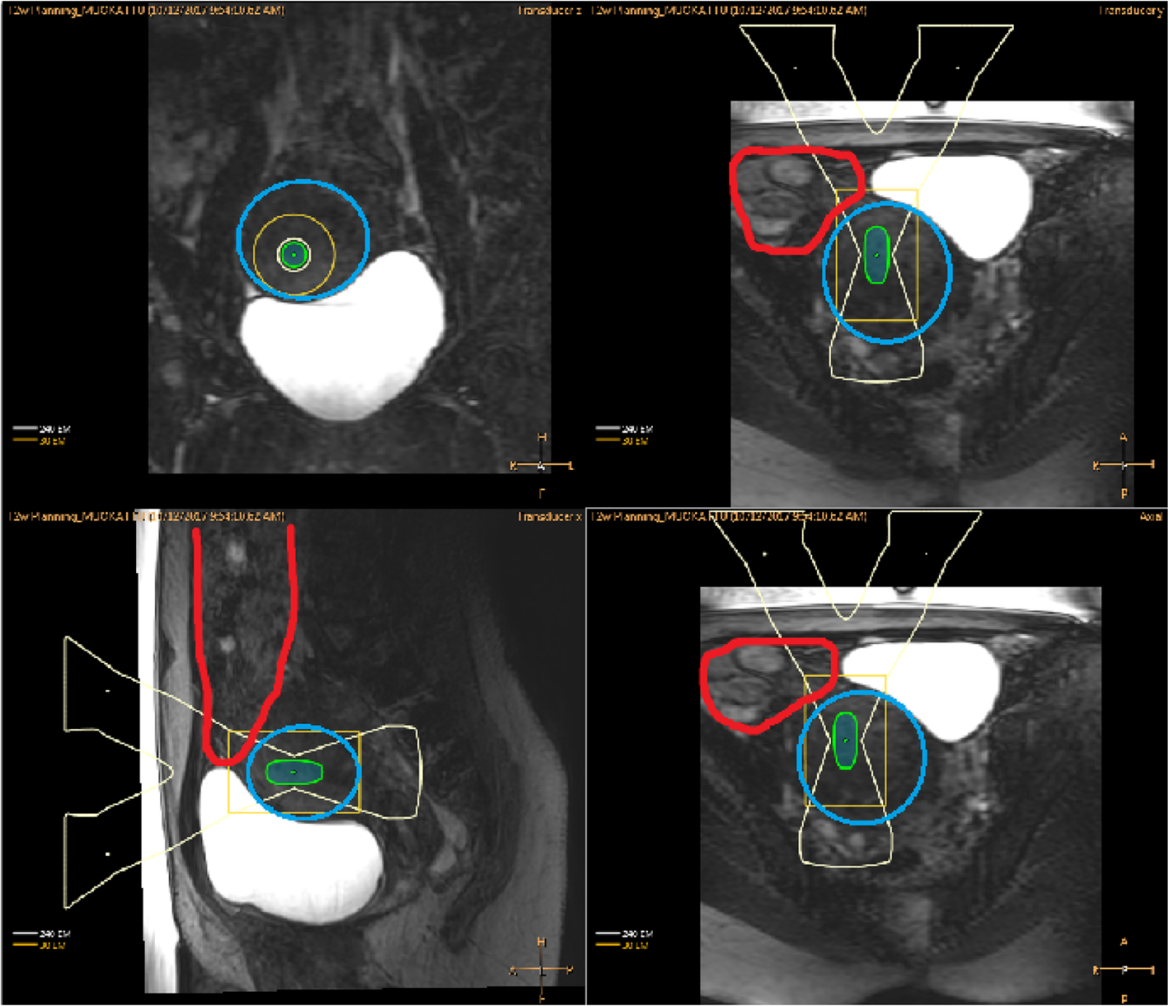
T2W planning images displayed on the therapy planning console with ultrasound beam view where bowels are presented with red color and target uterine fibroid not accessible with blue color

As demonstrated in Fig. 4, filling the urinary bladder to the point of discomfort did not achieve the desired results, and despite tilting and shaping of the ultrasound beam, sufficient coverage of the target could not be achieved (Fig. 4).

**Fig. 5:**
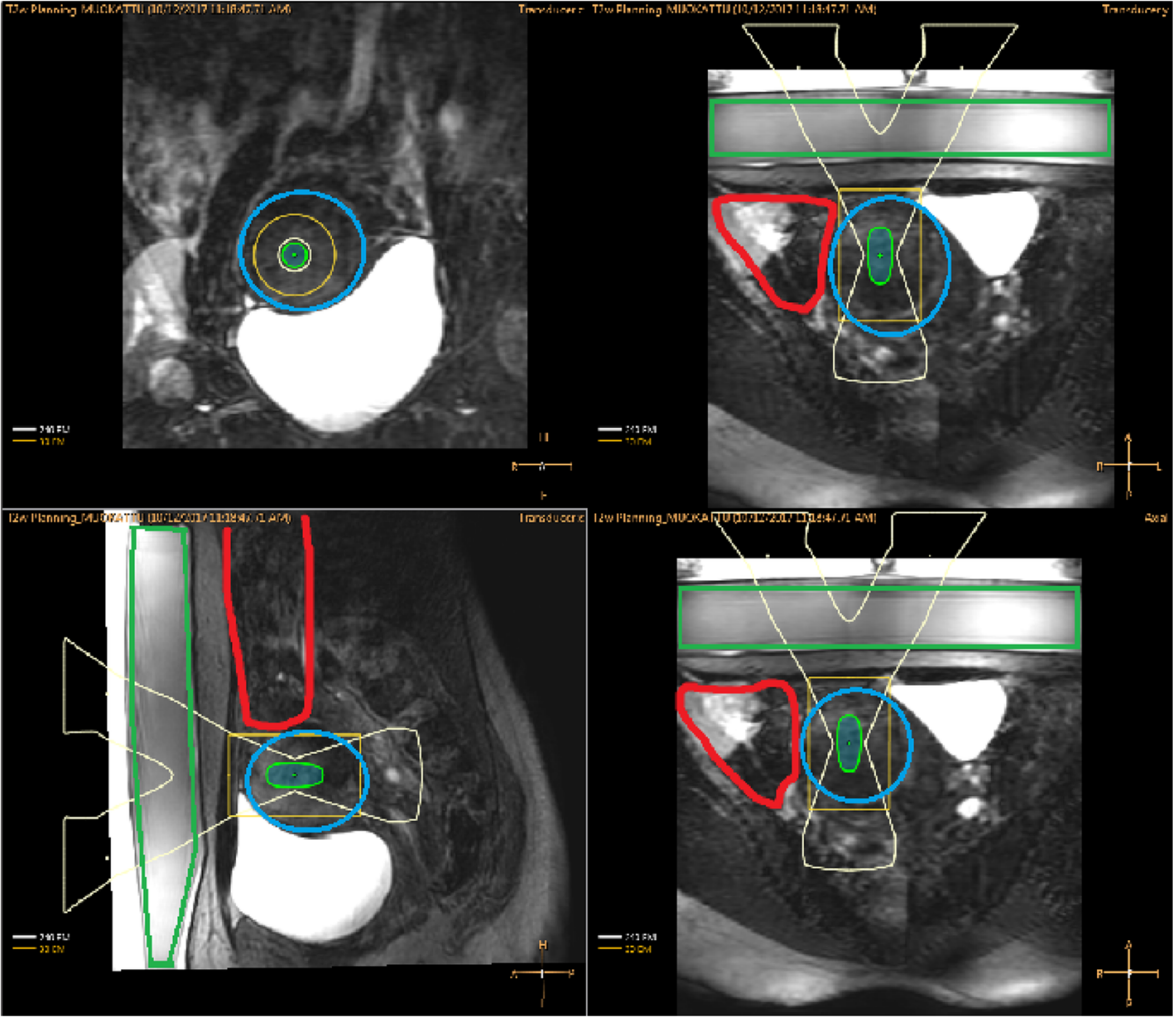
T2W planning images displayed on the therapy planning console with ultrasound beam view after application of wedged gel pad where red color boundary is bowels, blue is uterine fibroid, and green is the gel pad

After application of the wedged gel pad, the bowels were repositioned so that the two target fibroids could be safely sonicated (Fig. 5).

**Fig. 6:**
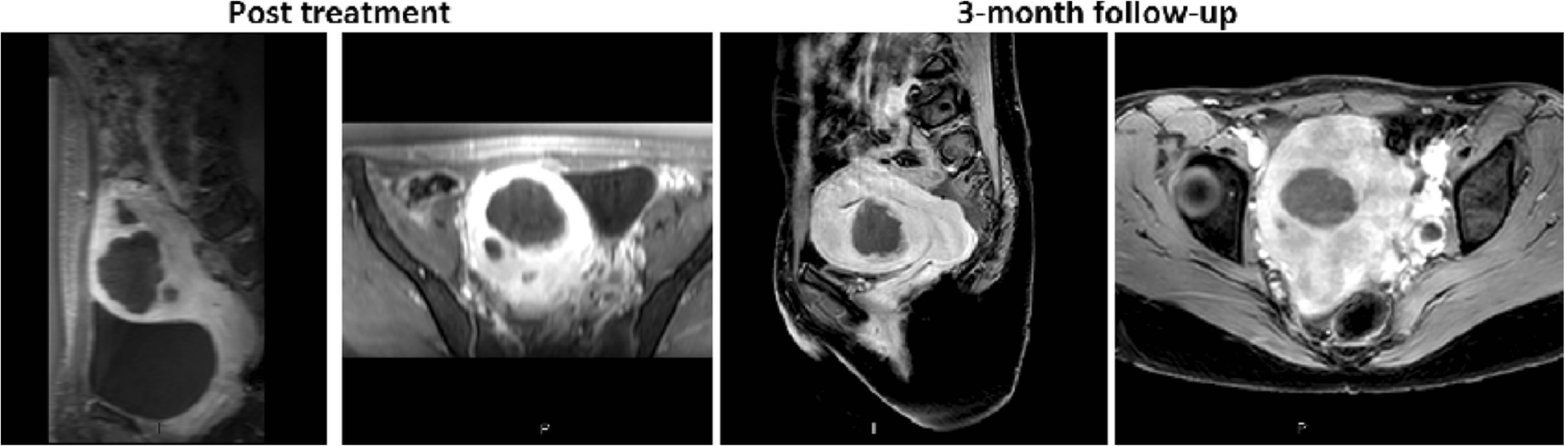
Contrast enhanced T1W images immediately after the treatment (NPVs: 86% and 39%) and at 3-month follow-up (Shrinkages: 49% and 2.5%)

Before therapy, good acoustic coupling between the surfaces was ensured with T1W sequence for detecting any air bubbles in the sonication path. The largest fibroid was treated first followed by the smaller fibroid. Good temperature rise was observed, average maximum temperature per sonication was 76.8^∘^C (63.8−115.1^∘^C), in both fibroids. The patient experienced mild pain during the treatment and opioid pain medication (fentanyl, 0.5 *μ*g) was administered. The patient reported the gel pad was getting hot during the sonications but no adverse effects were observed due to the treatment and gel pad usage. This highlighted the importance of increased cooling times between sonications. The total treatment time from first to last sonication (19 sonications) was 178 min and average treatment power and energy per sonication were 184 W and 5.3 kJ, respectively. Immediately after the treatment contrast-enhanced T1W images were acquired, indicating non-perfused volume ratios (NPVs) of 86% and 39% (Fig. 6). There were no areas of abnormal enhancement within the subcutaneous tissue, and skin was normal after treatment. The volume and shrinkage of the fibroids at 3 months follow-up were 29 mL and 49%, 5 mL and 2.5% respectively (Fig. 6).

#### Case 2

**Fig. 7:**
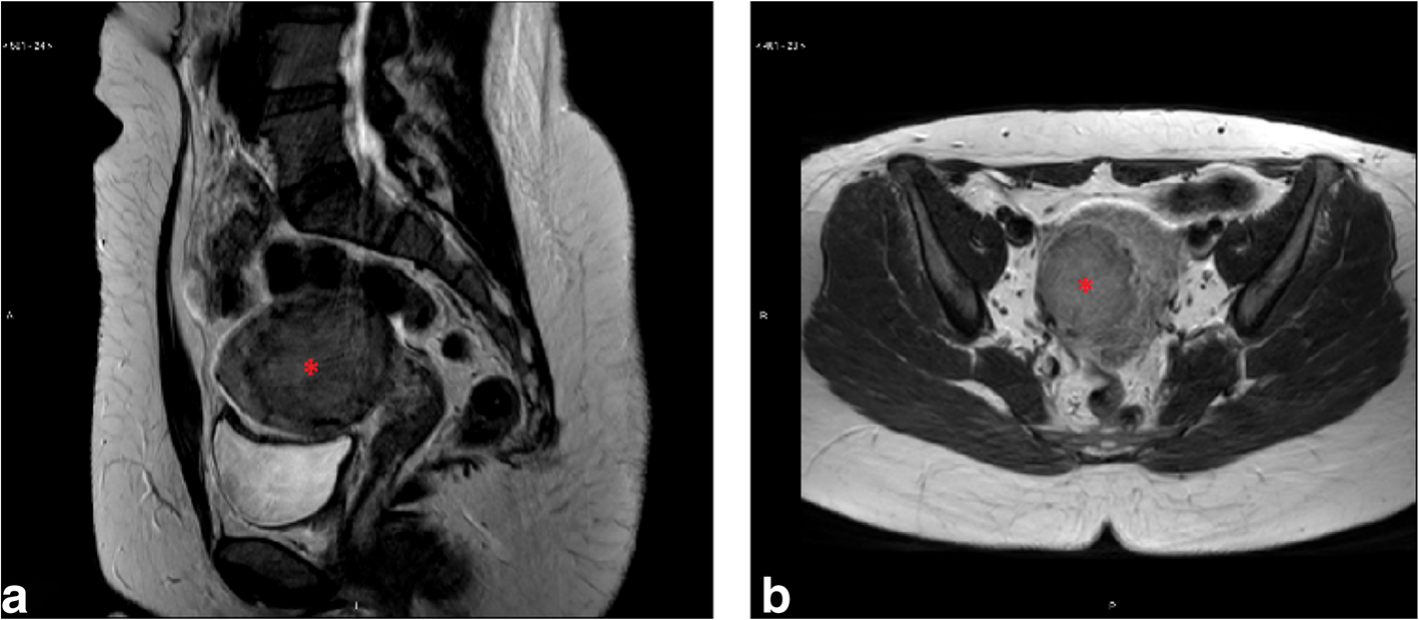
T2W screening images of uterine fibroid (red asterisk) in **a**) the sagittal and **b**) axial plane

A 40-year-old woman presented with symptomatic intramural uterine fibroid in posterior wall. The presented symptom was severe menorrhea. The fibroid was classified as Funaki type II based on T2W images, and contrast-enhanced T1W images were assessed. Fibroid size was 5.7 × 5.0 × 5.0 cm^3^ (Fig. 7).

**Fig. 8:**
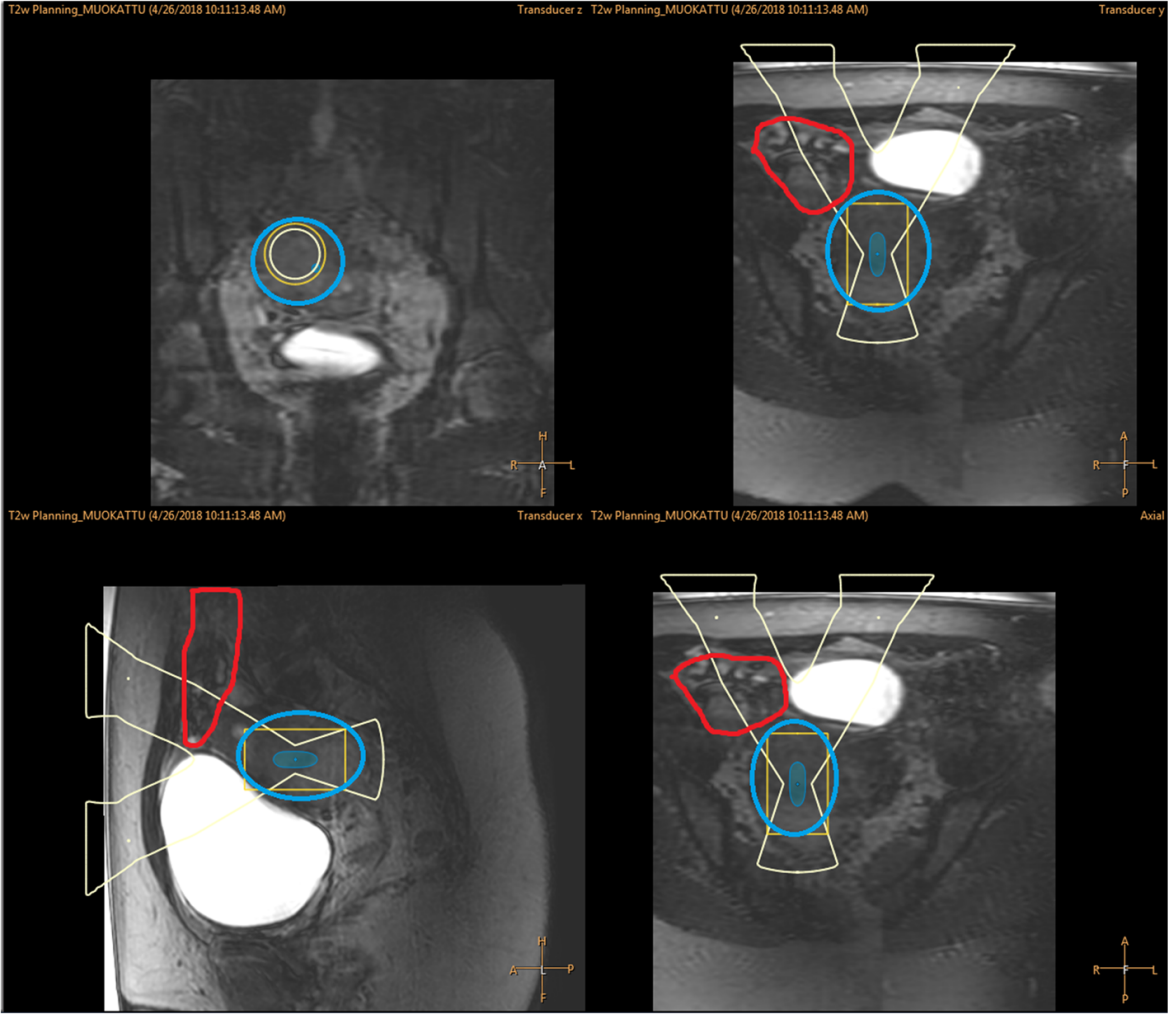
T2W planning images displayed on the therapy console with ultrasound beam view where bowels are presented with red boundary and uterine fibroid with blue boundary

As demonstrated in Fig. 8, the filling of the urinary bladder to the point of discomfort, again did not achieve the desired results and despite tilting and shaping of the ultrasound beam, sufficient coverage of the target could not be achieved (Fig. 8).

**Fig. 9:**
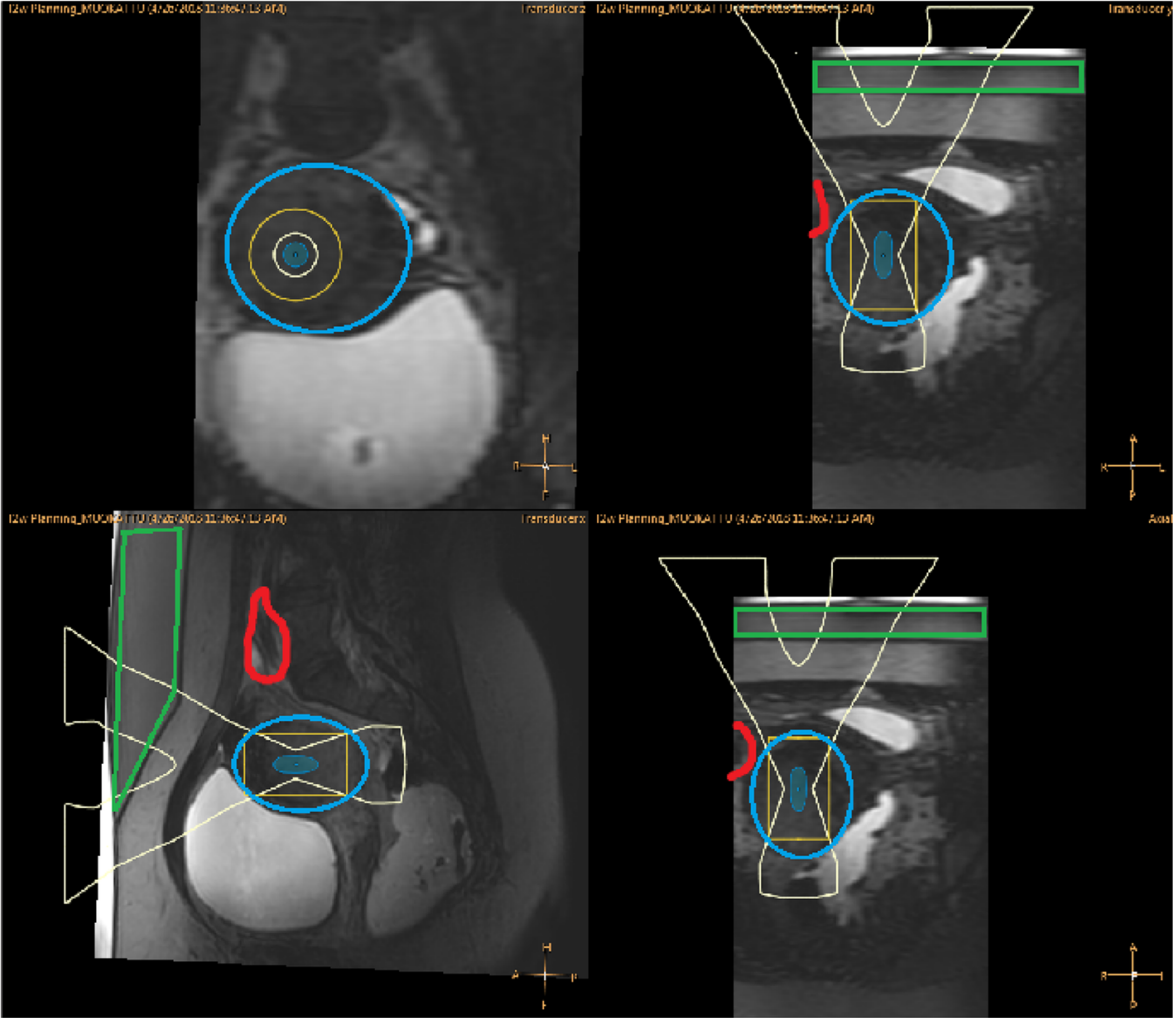
T2W planning images displayed on the therapy console with ultrasound beam view after application of wedged gel pad where red boundary is bowel, blue is uterine fibroid and green is the gel pad

After application of the gel pad, the bowels were repositioned so that the uterine fibroid could be targeted safely (Fig. 9).

**Fig. 10:**
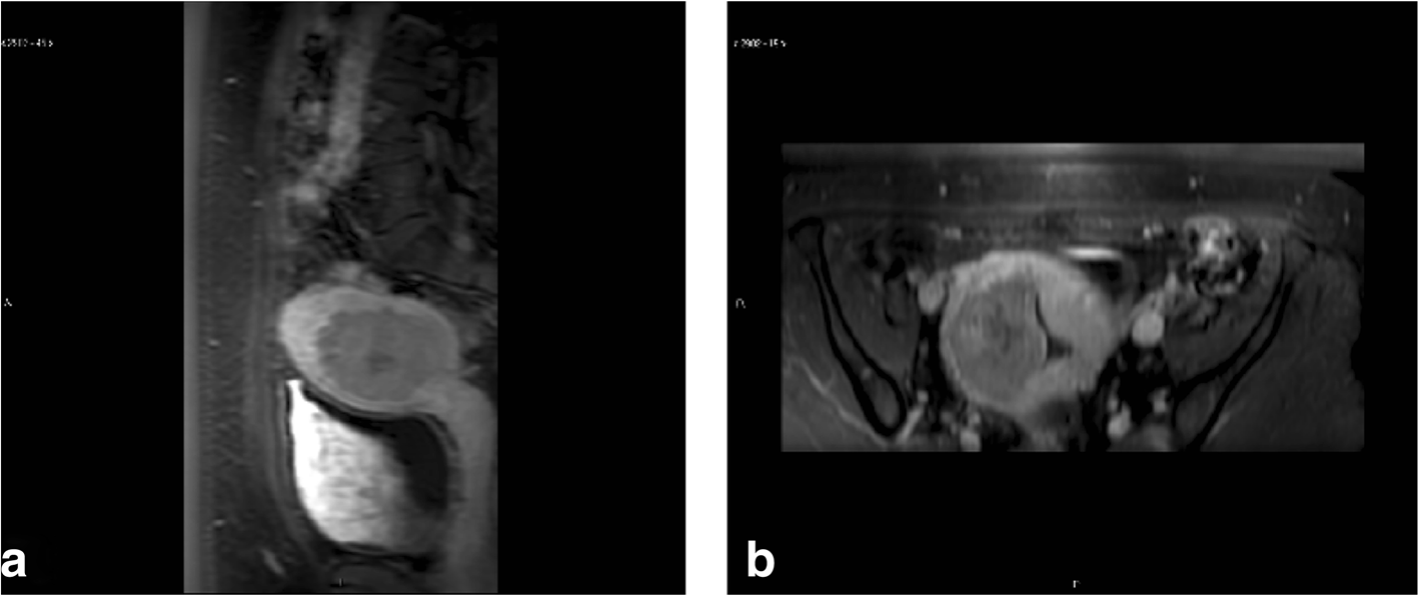
Contrast enhanced T1W images in **a**) sagittal and **b**) axial plane immediately after the treatment (NPV: 3%)

Before therapy, good acoustic coupling between surfaces was confirmed with T1W sequence to detect any air bubbles in the sonication path. Despite high power (300 W), a poor temperature rise was observed, average maximum temperature per sonication was 56.4^∘^C (47.3−72.4^∘^C) in the fibroid. The patient experienced severe pain during the treatment and opioid pain medication (fentanyl, 0.5 *μ*g) was administered twice during the treatment. The patient reported a sensation of heat on the skin surface during the sonications and after the treatment the skin looked visually irritated due to the treatment and the gel pad usage. The total treatment time from first to last sonication (9 sonications) was 155 minutes and average treatment power and energy per sonication were 284 W and 6.8 kJ, respectively. Immediately after the treatment, contrast enhanced T1W images were acquired, showing non-perfused volume ratio (NPV) of only 3% (Fig. 10). For this reason more detail analysis was done after the treatment. The conclusion is that the poor treatment result was most likely caused by high perfusion in the fibroid which prevents the tissue from reaching the thermal ablation temperature. This conclusion was reached based on dynamic contrast enhanced images that were obtained during the screening MRI. There were no areas of abnormal enhancement within the subcutaneous tissue, however there was reddening of the skin which disappeared in few hours. This could be caused by the gel pad as it is functioning as an insulator and the cooling effect of direct skin cooling device does not reach the skin as effectively as without the gel pad. This results in higher tissue temperatures which may cause irritation to the skin or even skin burns if cooling times are not sufficient.Since this is unusual shape of the gel pad in not taken into account by the software, maybe we should use slightly longer cooling times in these cases. Due to poor treatment result patient was settled on hysterectomy.

## Discussion

In MR-HIFU treatments, unfavorable bowel loop position can prevent or limit the treatment window, and therefore, also the volume where HIFU therapy can be delivered to. Manipulation of the bowel loops with bladder and/or rectum filling can be attempted but these are not always sufficient to reposition the bowel loops. Hesley et al. [25] reported that a convex gel pad was helpful in displacing the bowel loops from the treatment window during HIFU therapy.

In this report, we assess MR-HIFU therapy of two patients with a total of four symptomatic uterine fibroids where bladder and/or rectum filling was not sufficient to reposition the bowel loops. Temperature images were closely monitored to detect any unwanted heating on the gel pad surface. Longer cooling times were used during the gel pad assisted treatments to account for cumulative heating on the skin, due to gel pad preventing the cooling effect to the skin from the direct skin cooling device. The basic principle was that the more power was used the longer cooling time was applied. The minor adverse effects in the second patient with Funaki type II fibroid could suggest that the use of wedged gel pad is not preferable in treating Funaki type III fibroids and that care should be taken when treating Funaki type II fibroids. However, there were no thermal injuries in the abdominal muscle or subcutaneous fat layer.

Poor treatment result in case two demonstrates that Funaki classification alone may not be sufficient to predict treatment outcome. Particularly in Funaki type II fibroids. In these cases perfusion and diffusion imaging might offer additional, sometimes crucial, information.

Both MR-HIFU therapies were technically successful. The wedged gel pad in the sonication beam did not seem to reduce the efficacy of the energy delivery to the target, which was expected as the gel pad mostly consists of water. Due to the asymmetric shape of the gel pad in the ultrasound beam path, a slight deformation in the ultrasound focal point might be induced. However, the patient-specific ultrasound simulations were conducted and they showed that the use of the wedged gel pad does not cause a reduction in the ultrasound peak pressure nor focal point deformation, and therefore, no therapeutic efficacy is lost.

The wedged gel pad seems to be able to create a force in anteroposterior direction which increases the pressure in the lower abdomen displacing bowel loops in cranial direction allowing a safe sonication path to the fibroid. Wedged gel pad can therefore be used as an alternative or adjuvant bowel repositioning technique. Using this technique the number of patients suitable for MR-HIFU treatment could be increased, and better treatment results could be achieved in cases where bowel loops limit the treatment window. On the other hand there are also drawbacks related to this technique, for example increased cumulative heating which can result in skin irritations. It might also be more time consuming than bladder and/or rectum filling techniques.

Among the limitations of this study have to be mentioned the small number of patients and the lack of Funaki type III fibroids, although these type of lesions are typically not well suitable for HIFU therapy [16].

## Conclusions

In conclusion, our experience presented here, suggests that the use of wedged gel pad during MR-HIFU treatment could be a simple and effective tool to manipulate the bowels in cases where filling of the bladder is not sufficient to create a suitable treatment window. Wedged gel pad could also be beneficial in other situations to achieve better treatment coverage to the uterine fibroid.
